# Effect of pomegranate juice on paraoxonase enzyme activity in patients with type 2 diabetes

**DOI:** 10.1186/2251-6581-11-11

**Published:** 2012-08-31

**Authors:** Nayereh Parsaeyan, Hassan Mozaffari–Khosravi, Mohammad Reza Mozayan

**Affiliations:** 1 Department of Biochemistry, Shahid Sadoughi, University of Medical Sciences, Yazd, Iran; 2 Department of Nutrition, Shahid Sadoughi University of Medical Sciences, Yazd, Iran; 3 Department of English Language, Shahid Sadoughi University of Medical Sciences, Yazd, Iran; 4 Department of biochemistry, School of medicine, Shahid Sadoughi University of Medical Sciences, Yazd, Iran

**Keywords:** Pomegranate juice, PON1 activity, Diabetes mellitus

## Abstract

**Objective(s):**

Paraoxonase-1 (PON1), an HDL-associated enzyme, prevents lipoprotein oxidation. PON1 enzymatic activity has been shown to decrease in patients with diabetes. Paraoxonase activity. HDL capacity to bind with PON1 is possible under specific experimental conditions, such as oxidation, addition of polyphenols, or in diabetic patients with polyphenols doses. The aim of this study was the effect of pomegranate juice (PJ) on paraoxonase and arylesterase activity of PON1.

**Materials and methods:**

Fifty patients with type 2 diabetes mellitus consumed 200 ml of PJ daily for a period of 6 weeks. Blood was collected from the patients before and after PJ consumption after 12 h of fasting. Blood sugar, total cholesterol, triglyceride, LDL- C and HDL-C were measured by colorimetric kit method. The malondialdehyde concentration (μmol/L) was determined by thiobarbituric acid (TBA) assay. Paraoxonase and arylesterase activity of PON1 enzyme were measured using paraoxone and phenylacetate as the substrates.

**Results:**

The concentration of fasting blood sugar, total cholesterol, LDL-C and malondialdehyde significantly (p < 0.001) decreased after the intervention. Paraoxonase and arylesterase activity of PON1 significantly (p < 0.001) increased after the intervention. There were however no significant changes in serum triglyceride and HDL-C. There was a significant positive correlation between paraoxonase and arylesterase activity of PON1 and serum HDL-C concentration . A significant negative correlation was detected between paraoxonase and arylesterase activity of PON1 and FBS.

**Conclusion:**

It can be concluded that PJ consumption as an antioxidant may have a contribution in changing fasting blood sugar, lipid profiles, lipoprotein oxidation, and PON1 activity.

## Introduction

Paraoxonase (EC.3.1.8.1, aryldialkylphosphatase) has been extensively studied in the field of toxicology [1]. Paraoxonase hydrolyzes organophosphate compounds, are widely used as insecticides and nerve gases [2,3]. Human serum paraoxonase (PON1) is synthesized in the liver and is physically associated with HDL, on which it is almost exclusively located. Several studies have indicated that PON1 can prevent lipid peroxide accumulation on LDL both in vitro and in vivo [4,5]. Some studies have shown that serum PON1 activity is reduced in diabetes [6-17]. Recently, studies in PON1 demonstrated that PON1 has a protective role against diabetes development, secondary to its unique antioxidant properties [13]. The high concentrations of glucose in diabetic serum could account for PON1 dissociation from HDL [14]. Paraoxonase activity is under both genetic and environmental influences and varies widely among individuals [11].

Among the main risk factors responsible for coronary heart disease (CHD), diet has an important role in patients with diabetes as well, because it regulates the levels of plasma lipids and lipoproteins, blood pressure, energy balance, thrombogenesis, and the oxidative modification or protection of plasma lipids and lipoproteins [15]. PJ contains polymolecular ellagitannin compounds, such as punicalagin, which is a potent antioxidant [16,17]. Recent findings have demonstrated that consumption of PJ by patients with diabetes decreases oxidative stress in their serum and contributes to PON1 stabilization, increases PON1 association with HDL, and stimulates the enzyme catalytic activities [18,19]. The aim of the present study was to determine the effect of PJ consumption on paraoxonase enzyme activity in patients with type 2 diabetes.

## Materials and methods

In this quasi-experimental interventional study, fifty patients with type 2 diabetes mellitus, consumed 200 ml of PJ daily for a period of 6 weeks. The patients served as their own controls because we compared all data after the pomegranate consumption with the baseline values. The patients in the study had no hypertension and cardiovascular diseases. None of the participants received any antioxidant supplementations in the past 3 months. They followed their own normal diet. PJ was prepared from the whole fruit after being cut and exposed to arils for the squeezing process. The juice was then filtered, pasteurized, concentrated and stored at −18°C. The concentrated PJ was diluted 1:4 (v:v) to 16 Brix (Brix is a measurement of soluble solids in fruit juice and represents the sugars and many other soluble substances such as salts, acids, and tannins. Brix is measured in grams per hundred milliliter) with water to obtain a single-strength PJ to be used in the study. Blood was collected from the patients before and after PJ consumption after 12 h of fasting. Serum was then separated, and fasting blood sugar (FBS), total cholesterol, triglyceride and HDL-C were measured by enzymatic and colorimetric kit method . The concentration of LDL-C was calculated by using Friedwald formula. The malondialdehyde concentration (μmol/L) was determined by thiobarbituric acid (TBA) assay. Paraoxonase activity (μmol/L) was measured by adding 20 μL serum to 700 μL buffer [2 mmol/L paraoxone, 2 mmol/L Cacl2 in 1 mmol/L Tris–HCl buffer (PH = 8)] and then Para-nitrophenyl as a product was measured at 412 nm wavelength. Also arylesterase activity of PON1 was measured by using 10 μL serum and mixture of 2 mmol/L phenylacetate and 2 mmol/L Cacl2 in 100 mmol/L Tris–HCl buffer (PH = 8). Then hydrolysis rate of phenylacetate at wavelength 270 nm was measured by spectrophotometer.

## Results

The ratio of male to female patients was nearly the same. The patients^,^ mean age was 45 ± 8y and the mean for their BMI was 30 ± 3 kg/m^2^ . Table 1 shows the comparison of serum lipids, lipoproteins and malondialdehyde before and after PJ consumption. As shown in Table 1 mean of FBS, total cholesterol, LDL-C and malondialdehyde decreased significantly (p < 0.001) after the intervention. There were however no significant changes in serum triglyceride and HDL-C concentration. Table 2 shows paraoxonase and arylesterase activity of PON1 and their ratio before and after PJ consumption. Para-oxonase and aryl esterase activity of PON1 and their ratio increased significantly after the intervention (p <0.001). Also the correlation between paraoxonase activity and arylesterase activity of PON1 and other biochemical parameters revealed a positive correlation between paraoxonase and arylesterase activity of PON1 and serum HDL-C concentration which was statistically significant. A significant negative correlation was detected between paraoxonase and arylesterase activity of PON1 and FBS (Table 3).

**Table 1:** Mean concentration and standard deviation of fasting blood sugar (FBS), lipids, lipoprotein and malondialdehyde in patients with type 2 diabetes before and after pomengranate juice consumption

**Biochemical parameters**	**Before**	**After**	**P-value**
	**Mean ± SD**	**Mean ± SD**	
FBS(mg/dl)	195.38 ± 31.91	160.64 ± 37.47	<0.001
TC(mg/dl)	179.02 ± 29.16	160.04 ± 11.98	<0.001
TG(mg/dl)	169 .64 ± 10.70	162.08 ± 9.02	0.05
HDL-C(mg/dl)	36.58 ± 4.60	38.36 ± 5.56	0.98
LDL-C(mg/dl)	101.29 ± 15.78	85.12 ± 13.94	<0.001
MDA(μmol/L)	0.073 ± 0.046	0.029 ± 0.021	<0.001

FBS = fasting blood sugar, TC = total cholesterol TG = triglyceride, HDL-C = high density lipoprotein cholesterol, LDL-C = low density lipoprotein cholesterol, MDA = malondialdehyde.

**Table 2:** Mean concentration and standard deviation of the paraoxonase activity,arylesterase activity of PON1 and their ratio in patients with type 2 diabetes before and after pomengranate juice consumption

**Enzyme activity**	**Before**	**After**	**P-value**
	**Mean ± SD**	**Mean ± SD**	
Paraoxonase activity (μmol/L)	135.02 ±104.14	225.18 ± 149.52	<0.001
Arylesterase activity (μmol/L)	165.02 ± 56.63	246.36 ± 49.26	<0.001
Aaraoxonase/arylesterase	1.22 ± 0.54	1.94 ± 0.32	<0.001

**Table 3:** Correlation between paraoxonase activity and arylesterase activity Of PON1 and other biochemical parameters

**Biochemical parameters**	**Paraoxonase activity**		**Arylesterase activity**	
	**r**	**p-value**	**r**	**p-value**
FBS(mg/dl)	−0.690	0.000	−0.718	0.000
TC(mg/dl)	−0.234	0.270	−0.090	0.67
TG(mg/dl)	−0.071	0.74	−0.054	0.80
HDL-C(mg/dl)	0.451	0.027	0.622	0.001
LDL-C(mg/dl)	−0.245	0.249	−0.040	0.854
MDA(μmol/L)	−0.432	0.021	−0.502	0.035

## Discussion

Pomegranate is an important source of bioactive compounds and has been used in traditional medicine for centuries. PJ is known to be high in antioxidant activity [15-19]. The present study was designed to evaluate the effect of PJ on FBS, lipid profiles, lipid oxidation PON1 paraoxonase activity, arylesterase activity of PON1 and their correlation in patients with type 2 diabetes. Our results showed that daily consumption of 200 ml PJ decreased the mean of FBS, total cholesterol, LDL-C and malondialdehyde significantly (p <0.001). There were however no significant changes in serum triglyceride and HDL-C concentration. The results of this study are in line with that of M.I. Gil et al. They reported that consumption of PJ in patients with type 2 diabetes decreases cholesterol and LDL-C concentration [15]. Esmailzadeh A. et al. have shown significant reduction of total cholesterol (P <0.006), LDL-C (P <0.006), LDL-C/HDL-C (P <0.001), and total cholesterol/HDL-C (P <0.001) after 8 weeks of PJ consumption in patients with diabetes. There were however no significant changes in serum triacylglycerol and HDL-C concentrations [16].

In this study, mean of paraoxonase and aryl esterase activity of PON1 and their ratio were increased significantly after the intervention (p < 0.001). This indicated that polyphenols compounds in PJ have antioxidant effect. Rosenblat. M. et al. reported that PJ consumption results in a significant reduction of thiobarbituric acid reactive substances (TABARS) and an increase in PON1 activity thus being in line with that of our study [18,19]. Yukio Ikeda et al. and other investigators have shown that PON1 levels decrease in patients with diabetes [6-17].

PJ increased PON1 activity because its components(tannins and anthocyanin) have direct effect on enzyme activity [15]. In this study the correlation between paraoxonase activity and arylesterase activity of PON1 and other biochemical parameters showed that there is significant positive correlation between paraoxonase and arylesterase activity of PON1 and serum HDL-C concentration (r = 0.451 p = 0.027, r = 0.622 p = 0.001). Also there is a significant negative correlation between paraoxonase and arylesterase activity of PON1 and FBS(r = 0.69 p < 0.001, r = 0.718 p < 0.001). Measurment of PON1 enzyme activity, evaluation of changes in its performance in patients with type 2 diabetes and managing PJ supplement were the new and positive points of this research. Measurment of just one antioxidant enzyme was the negative aspect of the study. Therefore more extensive studies have to be carried out on PON1 enzyme for the management of diabetes.

## Conclusion

These results demonstrated that PJ consumption for 6 weeks may exert beneficial effects on fasting blood sugar, lipid profiles, lipoprotein oxidation and PON1 activity. The juice therefore can have more potential as a health supplement rich in normal antioxidant.

## Competing interests

The authors declare that they have no competing interests

## Authors' contribution

N Parsaeyan and Dr H Mozaffari–Khosravi are scientific members of Biochemistry and nutrition department, respectively. They designed and supervised this study. MR Mozayan made a comprehensive revision of the article. All authors read and approved the final manuscript.
